# Mass Spectrometry Imaging as a Tool to Investigate Region Specific Lipid Alterations in Symptomatic Human Carotid Atherosclerotic Plaques

**DOI:** 10.3390/metabo11040250

**Published:** 2021-04-18

**Authors:** Francesco Greco, Laura Quercioli, Angela Pucci, Silvia Rocchiccioli, Mauro Ferrari, Fabio A. Recchia, Liam A. McDonnell

**Affiliations:** 1 Institute of Life Sciences, Sant’Anna School of Advanced Studies, 56127 Pisa, Italy; fr.greco@santannapisa.it (F.G.); f.recchia@sssup.it (F.A.R.); 2 Fondazione Pisana per la Scienza ONLUS, 56017 San Giuliano Terme (PI), Italy; 3 Department of Vascular Surgery, Azienda Ospedaliero Universitaria Pisana, 56124 Pisa, Italy; lauraquercioli08@gmail.com (L.Q.); mauro.ferrari@unipi.it (M.F.); 4 Department of Histopathology, University Hospital, 56124 Pisa, Italy; angelapucci@libero.it; 5 Institute of Clinical Physiology, National Research Council, 56124 Pisa, Italy; silvia.rocchiccioli@ifc.cnr.it; 6 Cardiovascular Research Center, Lewis Katz School of Medicine, Temple University, Philadelphia, PA 19140, USA

**Keywords:** MALDI MSI, atherosclerosis, plaque outcome, lipids, macrophages, vascular smooth muscle cells (VSMCs)

## Abstract

Atherosclerosis is characterized by fatty plaques in large and medium sized arteries. Their rupture can causes thrombi, occlusions of downstream vessels and adverse clinical events. The investigation of atherosclerotic plaques is made difficult by their highly heterogeneous nature. Here we propose a spatially resolved approach based on matrix-assisted laser desorption/ionization (MALDI) mass spectrometry imaging to investigate lipids in specific regions of atherosclerotic plaques. The method was applied to a small dataset including symptomatic and asymptomatic human carotid atherosclerosis plaques. Tissue sections of symptomatic and asymptomatic human carotid atherosclerotic plaques were analyzed by MALDI mass spectrometry imaging (MALDI MSI) of lipids, and adjacent sections analyzed by histology and immunofluorescence. These multimodal datasets were used to compare the lipid profiles of specific histopathological regions within the plaque. The lipid profiles of macrophage-rich regions and intimal vascular smooth muscle cells exhibited the largest changes associated with plaque outcome. Macrophage-rich regions from symptomatic lesions were found to be enriched in sphingomyelins, and intimal vascular smooth muscle cells of symptomatic plaques were enriched in cholesterol and cholesteryl esters. The proposed method enabled the MALDI MSI analysis of specific regions of the atherosclerotic lesion, confirming MALDI MSI as a promising tool for the investigation of histologically heterogeneous atherosclerotic plaques.

## 1. Introduction

Atherosclerosis is a chronic inflammatory disease [1] characterized by the accumulation of a fatty or fibro-fatty plaque extending from the intima to the media layer of large and medium-sized arteries [2,3]. Atherosclerosis is responsible for more than two thirds of deaths due to cardiovascular diseases [4]. Atherosclerotic plaque formation is a complex series of events that occurs in multiple stages. The lesion is initiated by the accumulation of low density lipoproteins (LDL) in the subendothelial matrix [3], that cause inflammation and monocyte recruitment from the bloodstream [5]. Monocytes differentiate into macrophages which internalize modified LDL. The inability to export the cholesterol derived from LDL [5] causes the death of the macrophages, which then accumulate in a lipid-rich necrotic core that sustains inflammation [6]. In addition to this, vascular smooth muscle cells (VSMCs) from the media layer proliferate, migrate and differentiate to produce a fibrous cap that encapsulates the lesion [7,8]. Thus, the atherosclerotic plaque is a complex and heterogeneous environment, formed by different populations of cells that interact with each other.

The major threat of the atherosclerotic plaque is the rupture or erosion of the fibrous cap, and the subsequent release of material into the bloodstream. Plaque rupture triggers the formation of thrombi that can clog downstream vessels, causing strokes or infarctions [8]. Plaque vulnerability originates from the biology of the lesion [9]. Inflammation, the balance between collagen synthesis and degradation, macrophage number and the dimension of the lipid core are some of the factors that have been reported to influence plaque stability [9]. Nevertheless, the biological mechanisms that cause plaque vulnerability are not yet fully understood [10]. Plaque rupture can provoke damage in distal tissues and lead to major symptoms. In carotid atherosclerosis the most common symptoms include transient ischemic attack (TIA), amaurosis fugax and minor strokes [11]. The appearance of such neurological symptoms in patients with highly stenotic plaques is an indication of plaque embolization [12], and these patients are recommended for surgery [13]. The presence of symptoms is thus a sign of a rupture-prone plaque and an indirect indicator of plaque instability. A focus on plaque symptomatology could thus reveal the biological mechanisms underlying plaque fate.

Any molecular investigation of plaque stability needs to take into account the intrinsic heterogeneity of the plaque. Mass spectrometry imaging (MSI) is gaining interest for the study of atherosclerosis [14] because of its ability to simultaneously record the distributions of hundreds to thousands of molecules directly from clinical tissue samples. MSI is based on the acquisition of spatially resolved mass spectra. Images representing the distribution of the analytes are then calculated from the intensities of the mass spectral peaks. Matrix-assisted laser desorption/ionization (MALDI) MSI is one of the most common MSI techniques, and is the method used here. In MALDI MSI, thin tissue sections of fresh frozen (or formalin fixed) tissues are cut using a cryostat (or microtome) and mounted onto a conductive glass slide. The tissue sections are then prepared for MALDI MSI by the careful deposition of a fine layer of the MALDI matrix [15]. When irradiated with a pulsed UV laser (nanoseconds pulse length, frequency 337–355 nm) the MALDI matrix absorbs the laser energy and leads to the efficient ablation and ionization of matrix and analytes from the tissue [15]. The MALDI MSI dataset is acquired in a raster pattern, in which a mass spectrum is recorded for each pixel of the raster [16,17]. MALDI MSI can be used to analyze peptides, proteins, lipids and glycans just by using different MALDI matrices and tissue preparation procedures [18]. Here we focused on lipids as they are one of the main drivers of atherosclerosis, due to their role as mediators of many mechanisms involved in plaque progression and stability, including inflammation, VSMC proliferation and immune cell activation [19]. MALDI MSI has been used to investigate the lipid profile of mouse [20,21,22,23,24] and rabbit [25,26] plaques. A more systematic approach has been pursued [27] to investigate the lipid fingerprint of carotid plaque regions, and it was recently expanded to report the lipid profiles of the necrotic core, fibrin, foam cells, erythrocytes and calcified regions in human advanced carotid atheromas [28]. Nevertheless, no systematic approach has been developed to target the differences in lipid composition of the same plaque region between symptomatic and asymptomatic plaques.

Here we propose a MALDI MSI methodology to investigate the lipid biology underlying plaque symptomatology. The approach is based on plaque segmentation by histology and immunofluorescence, to accurately demarcate plaque histomolecular heterogeneity, which is then used to compare the lipid profile of the same region in symptomatic and asymptomatic plaques. The application of the method to a preliminary dataset proved the effectiveness of the approach and allowed the detection of dysregulated lipid species in specific regions of symptomatic plaques.

## 2. Results and Discussion

### 2.1. Image Co-Registration and Region Definition

**Figure 1:**
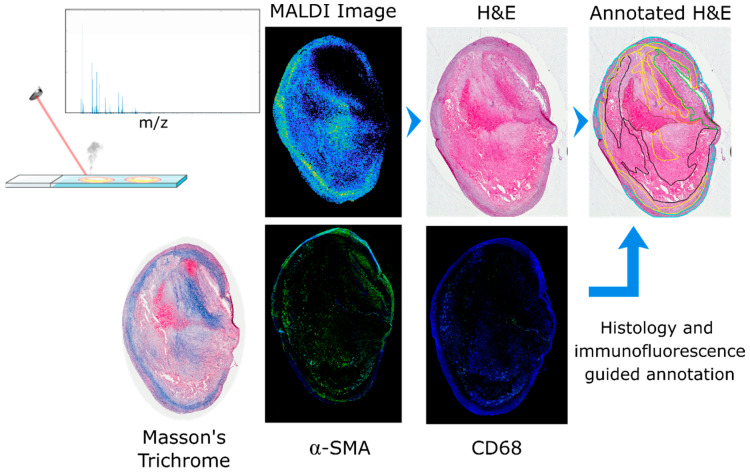
Annotation workflow for definition of regions. After MALDI MSI data acquisition, any residual matrix was removed and the tissue section histologically stained with hematoxylin and eosin. A consecutive section was stained with Masson’s trichrome to detect collagen (in blue). Two additional consecutive tissue sections were used for immunofluorescence of α-SMA and CD68 to detect VSMCs and macrophages, respectively. The histological image was then annotated based on the information gained from Masson’s trichrome and the immunofluorescence images; seven regions were defined: calcification (purple), hemorrhage (brown), lipid-necrotic core (black), collagen (yellow), macrophages (orange), outer VSMCs (cyan) and inner VSMCs (green).

Atherosclerotic plaques are very heterogeneous, composed of different cell types and regions. Biological processes within each region contribute to plaque stability and, eventually, rupture. For this reason accurate identification of each region and accurate co-registration of the MALDI MSI data to the histological image is essential. The histological images of the tissue sections were annotated on the basis of H&E and Masson’s trichrome staining supplemented with α-SMA and CD68 immunofluorescence images. Seven specific regions were defined: lipid-necrotic core, calcification, collagen-rich area, hemorrhage, macrophage-rich area, inner VSMCs and outer VSMCs. Outer VSMCs are defined as VSMCs of the media layer, and inner VSMCs are defined as VSMCs that have migrated in proximity to the lumen. Inner VSMCs are involved in the synthesis of extracellular matrix proteins of the fibrous cap that stabilize the lesion and are thereby important to plaque stability. Consecutive tissue sections were used to perform the assays needed to demarcate the tissue regions. Blue areas of the Masson’s trichrome staining were used to define collagen; immunofluorescence was used to identify macrophages (CD68 positive cells) and VSMCs (α-SMA positive cells). These annotations were then transferred to the histological image of the atherosclerotic plaques after checking cell morphology and region cellularity. The lipid-necrotic core, hemorrhage and calcification were annotated directly on the histological images (Figure 1).

**Figure 2:**
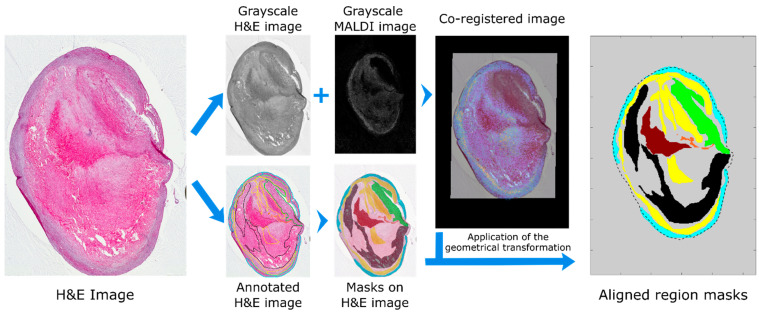
Workflow for transfer of molecular and histological annotations to MALDI MSI datasets. The histological images were imported into MATLAB, resized and rotated to approximately match the MALDI MSI datasets and saved as a grayscale image. The resulting image is co-registered to a user defined MS image and all parameters of the transformation saved. The coordinates of the annotated regions are imported into MATLAB and converted into masks with the command *poly2mask*. The same geometrical transformation used for the co-registration was then applied to register the region masks to the MALDI MSI dataset. Regions present in the plaque were hemorrhage (brown), lipid-necrotic core (black), collagen (yellow), macrophages (orange), outer VSMCs (cyan) and inner VSMCs (green).

Image co-registration was performed in MATLAB. High resolution histological images were imported into MATLAB, cropped, resized and rotated to better match the MALDI MSI dataset; the histological image was then converted to grayscale. A representative MS image from the MALDI MSI datacube was also converted to grayscale. The representative MALDI image corresponded to a high intensity ion co-localizing with the tissue section, an example of which is shown in Figure 2. The histological and MS images were then co-registered using an intensity-based co-registration algorithm (see methods for details). The transformation matrix, including initial cropping, rotation and scaling, was saved in a structured file for subsequent application to the annotation mask image. The annotation mask images were created from the border coordinates of the annotated regions using the command *poly2mask* (Figure 2). Co-registered masks were saved as a structured file for subsequent use during data analysis.

### 2.2. Comparison of DHB and Norharmane Matrices

We compared two MALDI matrices that are frequently used for lipid analysis [29], DHB and norharmane, to evaluate their performance with regard to the number of detected lipid ions and the ability of the lipid profiles to recapitulate the plaque’s histology. Furthermore, it is known that MALDI MSI can exhibit ionization bias, in which differences in the local chemical environment in heterogeneous tissues can lead to differences in the detection efficiency for particular analytes [30]. The use of two matrices was also used as a control, to guard against artifacts due to ionization bias.

**Figure 3:**
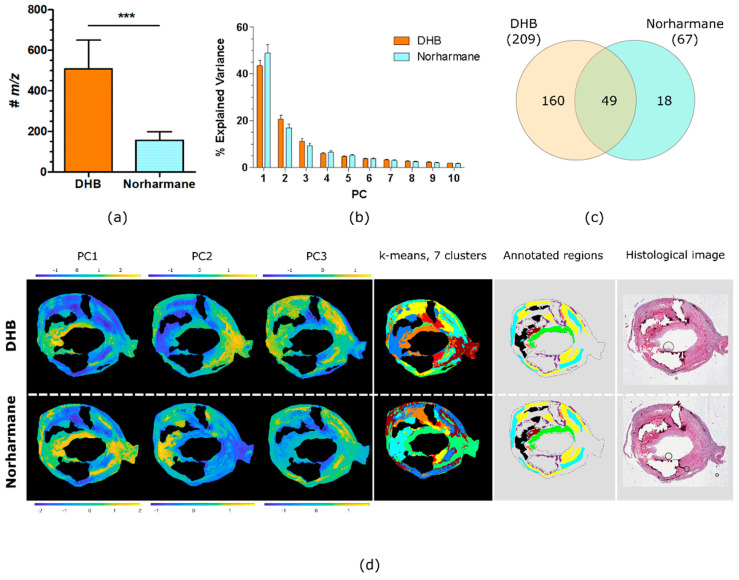
Comparison of DHB and norharmane matrices. (**a**) Number of lipid ions detected in all MSI datasets from all patients, for MSI datasets acquired using DHB and norharmane matrices (mean ± SD, two-tail paired *t*-test, ***: *p* < 0.001, *n* = 6). (**b**) Explained variance of the first ten PCs (mean ± SD, *n* = 6). (**c**) Venn diagram of the lipid ions in common between all MSI datasets acquired with DHB and norharmane. (**d**) Score images of the first 3 PCs, k-means clustering (7 clusters), annotated regions and histological images for the same carotid plaque. Annotated regions are calcification (purple), hemorrhage (brown), lipid-necrotic core (black), collagen (yellow), macrophages (orange), outer VSMCs (cyan) and inner VSMCs (green).

The number of discrete lipid ions detected with DHB and norharmane is shown in Figure 3a. A paired *t*-test demonstrated that DHB produced a significantly greater number of peaks compared to norharmane (509 vs. 157, *p* < 0.001, *n* = 6). Principal component analysis (PCA) was performed on each tissue section’s MSI data, for both matrices. The PCs generated by PCA maximize the variance in the data; the PCs capture the largest spread in the data and so effectively summarize its information content. The variance represented by the first ten principal components (PCs) is plotted in Figure 3b. The variance explained by the first PC of the DHB dataset was lower but was not statistically significant (paired *t*-test, *p* = 0.060, *n* = 6) compared to the variance explained by norharmane dataset. The DHB dataset contained 209 lipid ions that were detected in the tissue sections of all six patients; the norharmane dataset contained just 67 lipid ions common to all six patients, 49 of which were also common to the DHB dataset, Figure 3c and Supplementary Material 2.

The PCs generated by PCA of MSI data include scores for every pixel, and so can effectively summarize the distributions of the dominant lipid profiles in each tissue section. Figure 3d shows PCA score images for the first three PCs of a single plaque acquired with DHB and norharmane. A comparison of the score images with the annotations indicate that PC1 of the norharmane-prepared tissue section highlights VSMCs and PC2 correlates with the lipid-necrotic core. For the DHB-prepared tissue section, the score image of PC1 highlighted inner VSMCs (green annotated region) and PC2 outer VSMCs (cyan annotated region). Similarly, k-means cluster analysis of the MSI data demonstrated that inner VSMCs were defined in a unique cluster only in the DHB dataset. Image segmentation by k-means clustering (7 clusters) closely resembled the histological segmentation. Clusters corresponding to collagen and VSMCs were recognizable, with VSMCs further segmented in inner and outer VSMCs in the DHB dataset. The lipid-necrotic core, macrophage and hemorrhage regions were unified within a single cluster for both the DHB and norharmane datasets (Figure 3d).

The use of the DHB matrix led to MSI datasets containing a greater average number of ions correlating with the tissue’s histology than norharmane matrix. Moreover, the inner and outer VSMCs were distinguishable by PCA and k-means cluster analysis only in the MALDI MSI dataset acquired with DHB matrix. Thus, DHB performed better than norharmane. Nevertheless, the patient series was analyzed using both matrices to partially confirm, with the second matrix, lipid species found to be associated with the presence of symptoms, and thereby guarding against artifacts due to ionization bias.

### 2.3. Patient Series Analyses

To compare the same region across multiple patients, the MALDI MSI datasets from each patient were merged into a single datacube. ORBIIMAGEmzXML2Tricks, the software that performs peak picking and feature extraction of the mzXML MSI data, first produces a basepeak spectrum [31] of the MSI dataset. Then peak picking is performed on the basepeak spectrum and images corresponding to the selected features are extracted from the mzXML file and stored in a datacube. To compare datasets from different patient tissues it is important that the same *m/z* features are extracted from each patient’s individual MALDI MSI dataset. 

ORBIIMAGEmzXML2Tricks (v. 0.10, G. Eijkel) was modified to align the *m/z* axes of each dataset and then the basepeak spectra were combined to produce a global basepeak spectrum. Peak picking was then performed on the global basepeak spectrum and the resulting peak list used to extract the MS images from each sample’s dataset. In this manner, the resulting datacubes were derived from the same peak list, which includes all peaks detected in any of the tissue sections. Each MSI dataset was processed separately to remove any peak that did not exhibit a spatial correlation with the tissue, deisotoping the *m/z* list, and setting the intensity of pixels outside the tissue equal to zero. A project-specific merged datacube was produced by selecting only those peaks that were detected in all tissue sections being compared, and then using spatial offsets to place all datasets within the same coordinate space. The same spatial offsets were applied to the image mask files to produce project-specific mask for the merged dataset. If an annotated region was not present in a plaque, an empty mask of the correct dimensions was used in place of the missing region mask. In addition to the merged datacube, a list of lipid ions detected only in the asymptomatic plaques and only in the symptomatic plaques was determined. These lists, along with the *m/z* detected in each plaque, are available in Supplementary Material 6.

### 2.4. PCA of the Patient Series

PCA was applied separately to the DHB-matrix merged datacube and the norharmane-matrix merged datacube to compare the lipid profiles of the demarcated regions across all patient samples (Figure S7). In the DHB dataset, PC1 correlated most with collagen areas, except for patient 6 for which it also highlighted the lipid-necrotic core; PC2 correlated to VSMCs, especially from the inner layer, and with low scores for the lipid-necrotic core and macrophages. PC6 exhibited different correlations for asymptomatic and symptomatic plaques, namely with VSMCs for asymptomatic plaques and with macrophages and the lipid-necrotic core for symptomatic plaques.

PCA confirmed the compositional similarities of the same histological regions across the plaques of the dataset, but also indicated that there were differences between symptomatic and asymptomatic cases.

The consistency of the correlation of specific PC’s to specific histomolecular regions across the patient series confirmed that the same lipid species were sampled from each patient’s plaque and testifies to the similar composition of the plaque regions in different patients.

### 2.5. K-Means Cluster Analysis of the Patient Series

**Figure 4:**
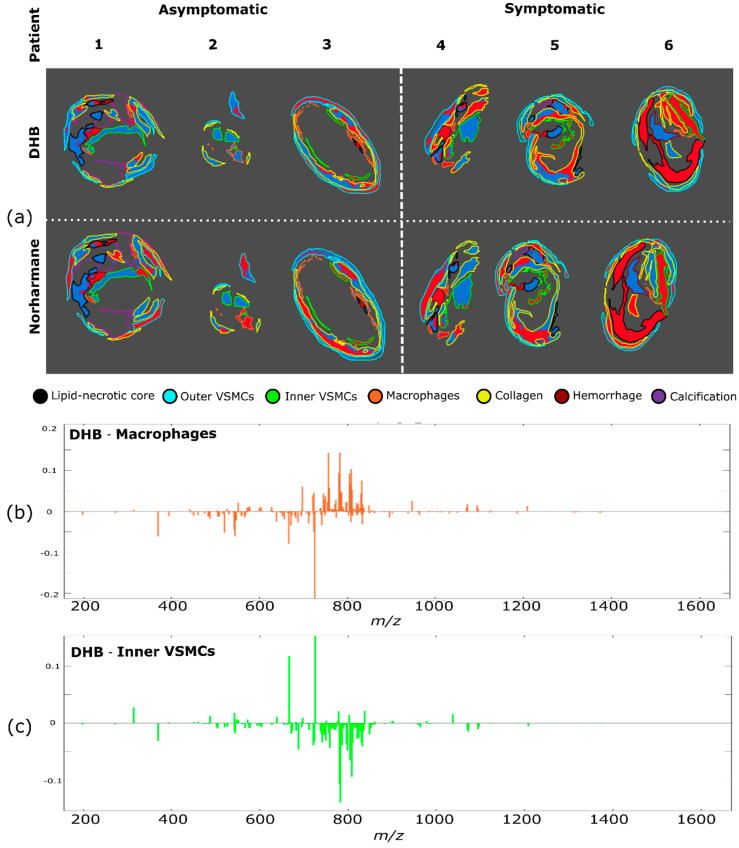
Cluster analysis for each region. (**a**) Pixels of each region were subjected to k-means clustering (two clusters for each region, red and blue) to discriminate between asymptomatic and symptomatic plaques. Region identity is coded by the border color. Clustering of different regions was performed independently. Difference between spectra of the blue and the red clusters (corresponding to cluster 1 and cluster 2) for (**b**) macrophages and (**c**) inner VSMCs regions of the DHB dataset.

To test whether areas from symptomatic plaques could be distinguished from the corresponding regions of asymptomatic plaques, we performed a region-specific cluster analysis, so that the significant differences in lipid composition between different regions did not mask any changes associated with plaque outcome. For each region, a k-means cluster analysis was performed with two clusters (Figure 4 and Figure S8) and the analysis was performed separately for the datasets measured with the DHB and norharmane matrices. Inner VSMCs and macrophage-rich regions both exhibited a dependence on the plaque outcome, with most pixels from symptomatic plaques clustering in cluster 2 (indicated in red) separately from asymptomatic plaques; inner VSMCs clusterization was consistent for datasets measured using both DHB and norharmane. The fate of macrophages has been linked to plaque outcome, with macrophage apoptosis increasing the size of the necrotic core, and thus helping sustain plaque inflammation and reducing plaque stability [6,32]. VSMC senescence and cell death have been linked to plaque vulnerability, and thus VSMCs have been postulated to have a crucial role in preventing plaque rupture [8,33,34].

### 2.6. PLS Regression of the Patient Series

We used a supervised approach to investigate the lipid ions associated with clinical status. We investigated separately the lipid ions that were detected only in the plaques from symptomatic and asymptomatic patients (Supplementary Material 6). One lipid ion was found exclusively in plaques from asymptomatic patients and two in symptomatic patients in the DHB dataset. Of these, only *m/z* 297.07, detected only in symptomatic plaques, could be assigned and was attributed to the sodiated adduct of glutamyl-glutamate. To further investigate the lipid ions associated with the clinical status of the patient, a PLS regression was performed on the ions detected in all plaques. An identical number of pixels were extracted from a defined region of each patient; the number of pixels used for the analysis corresponded to the largest number that could be extracted from all patients (see Methods for more detail). The ions with a VIP score greater than one and with a loading on the first PLS axis exceeding the 90th percentile of the positive or negative values are reported. The results for the macrophage region and the inner VSMCs are shown in Table 1 and Table 2, respectively; the results for the outer VSMCs, the lipid-necrotic core and the collagen region can be found in Supplementary Material 3.

**Figure 5:**
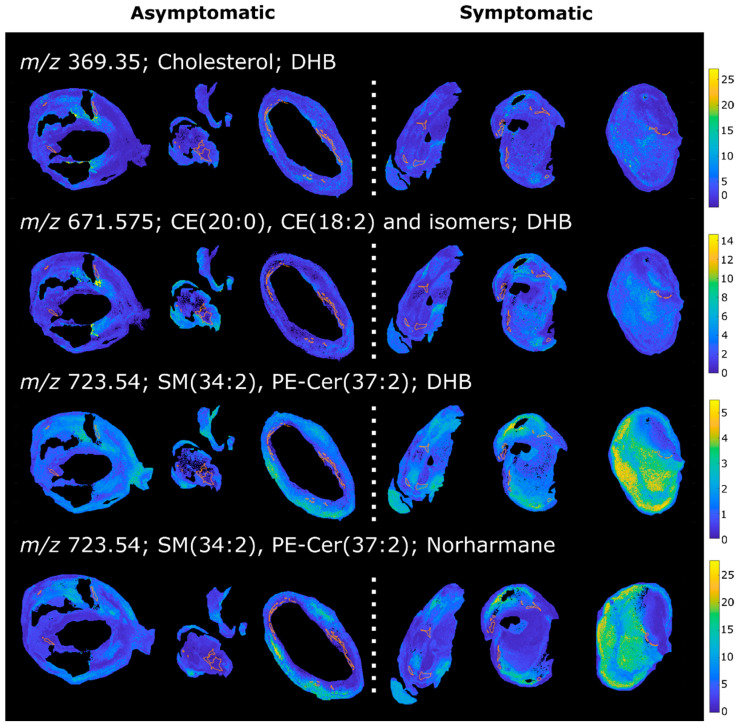
MALDI images of a selection of lipids upregulated in the macrophage area in symptomatic plaques. Macrophage-rich regions are highlighted.

Macrophage regions from symptomatic plaques were found to have higher levels of lipid ions that were assigned as sphingomyelins (SM) or phosphatidylethanolamine ceramides (PE-Cer). These lipids were detected in both the DHB and the norharmane datasets as sodiated adducts. Macrophage regions of symptomatic plaques also exhibited reduced levels of many phosphatidylcholines (PC), detected as sodiated and protonated adducts in the DHB and norharmane images, respectively. Sphingomyelins can interact with cholesterol in plasma membranes and play an important role in lipid raft formation and in inflammation processes [35,36,37]. Inhibition of endogenous SM synthesis reduces the size of atherosclerotic plaques in rodents, suggesting a role of SM in lesion onset and development [38,39,40]. Plasma SM have been proposed as a risk factor for coronary artery disease and atherosclerosis in rodents [41] and humans [42]. In our dataset, SM (34:2) was detected at increased levels in macrophage regions in symptomatic plaques in both the DHB and norharmane datasets (Figure 5), and has been previously described as characteristic of the necrotic core [28]. SM are involved in cholesterol efflux from macrophages and plaque stabilization. It was shown that a reduction of plasma membrane sphingomyelin in macrophages increases cholesterol efflux, reduces inflammation and stabilizes the plaque by decreasing the lipid core and increasing collagen content in mice [43]. Our results also suggest a role of sphingomyelin in macrophage-rich regions in plaque instability. An analogous increase in sphingomyelins and decrease in phosphatidylcholines was also present in the lipid-necrotic core of symptomatic plaques (Supplementary Material 3). This result was expected, since macrophages contribute to the buildup of the lipid core as apoptotic foam cells. 

Macrophage regions in plaques from symptomatic patients were also found to have higher levels of cholesterol and cholesteryl esters (CE) (Figure 5), the form in which cholesterol is stored in foam cells [44,45]. The ion at 369.35 *m/z* corresponds to the [M-OH]^+^ ion of cholesterol, but could also arise from the in-source fragmentation of cholesteryl esters. These species are major constituents of the atherosclerotic plaque and were previously identified by MALDI MSI in rabbit [23] and human [28] lesions. Moreover, an increase in total cholesterol content of atherosclerotic plaque has been previously observed in carotid plaques from symptomatic patients [46]. Lysophosphatidylcholines (LPC) were also found upregulated in the macrophage regions of symptomatic plaques. LPC play a role in LDL uptake by macrophages and in inflammation [47,48], and have been reported to be localized in vulnerable regions of murine and human lesions [49].

Inner VSMCs of symptomatic plaques were found to be enriched in cholesterol and CE, as well as PC and PE, constituents of lipid droplets of foam cells [47,50]. VSMCs have been described as a source of CE-enriched foam cells in human [51] and mice [52] atheromas. Their apoptosis is detrimental to plaque stability, increasing the size of the necrotic core and reducing collagen and fibrous cap thickness [33]. Differently, outer VSMCs of symptomatic plaques did not exhibit the same increase in cholesterol and CE (Supplementary Material 3), as expected considering their different phenotype.

### 2.7. Hierarchical Cluster Analysis

**Figure 6:**
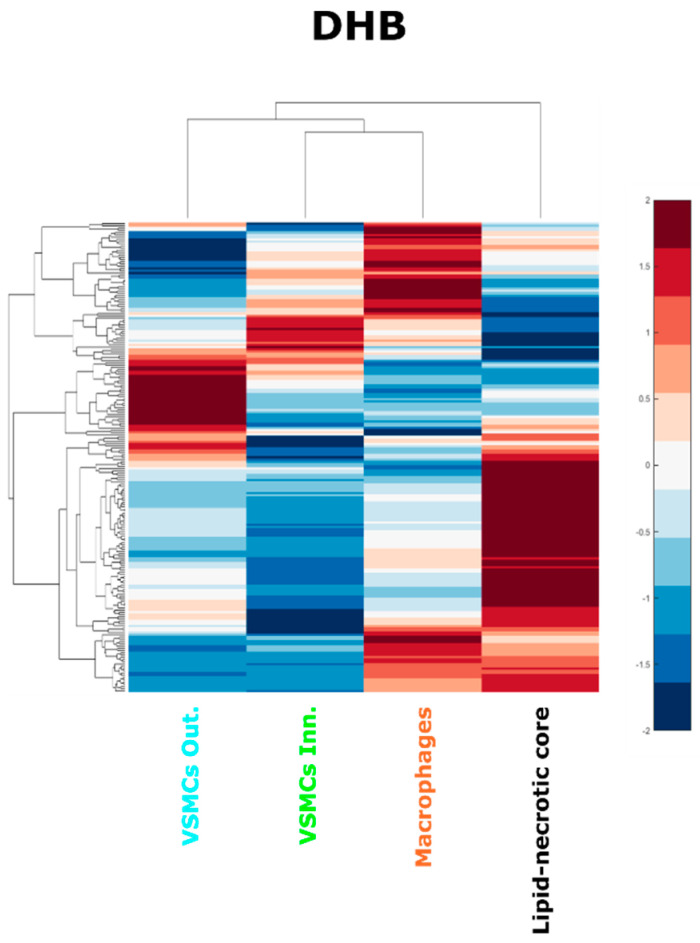
Hierarchical cluster analysis of the lipid-necrotic core, outer VSMC, inner VSMC and macrophage regions. Average spectra of the regions were used to perform a hierarchical cluster analysis for the DHB dataset.

During the evolution of atherosclerotic plaques different cell populations undergo phenotype switching and apoptotic processes. VSMCs in particular can lose their contractile phenotype, switching to a synthetic phenotype and even acquiring macrophage features, contributing to the foam cell population and ultimately to the lipid core [8]. Investigating the molecular similarities of the regions of the plaque could provide a more complete understanding of the cell interaction with the lipid-rich environment of the plaque. We thus performed a hierarchical cluster analysis of the average spectra from each region of each patient (Figure 6 and Figure S9).

The hierarchical cluster analysis confirmed that the composition of the lipid necrotic core is different from the other cell regions, presumably because of necrosis and the accumulation of cell debris from multiple sources. The hierarchical cluster analysis also revealed that the average lipid composition of the inner VSMC region resembled the macrophage region more than the VSMCs of the outer plaque layer. This result was consistent for MALDI-MSI datasets using both matrices (Figure S9). This finding is consistent with the hypothesis that inner VSMCs can share some characteristics with macrophages, including the internalization of modified LDL [52], the contribution of VSMCs to the foam cell population [52], and their expression of macrophage markers in late atheromas [8]. 

### 2.8. Study Limitations and Future Perspectives

Mechanisms underlying plaque fate towards stability or rupture are still largely unknown and their study is made difficult by the high heterogeneity of the atherosclerotic lesion. We propose to simplify the approach by focusing on specific regions of the plaque by using spatially resolved techniques. The proposed technique was applied to a small patient series to test its applicability. Preliminary results are promising but need to be confirmed in a larger dataset. The number of lipid species detected by MSI could be increased by using additional MALDI matrices, performing the analysis in negative ion mode as well as positive ion mode (as used here), and using the post-ionization methods of MALDI-2. Similarly, the regions indicated by MALDI-MSI as having molecular signatures that differentiate symptomatic from asymptomatic patients could be isolated with laser-capture microdissection and characterized by proteomics or RNAseq. Increased knowledge regarding the molecular mechanisms underlying plaque stability is needed for the development of improved pharmaceutical treatments, reducing the need for invasive carotid endarterectomy surgery.

## 3. Materials and Methods

### 3.1. Materials

2,5-Dihydroxybenzoic acid (DHB), norharmane, Fluoroshield with DAPI, Monoclonal Anti-Actin α-Smooth Muscle antibody, LC-MS grade methanol, HPLC grade chloroform, Bouin’s solution, Triton X-100, Tween 20, poly-L-lysine solution 0.1% and Peel-a-Way embedding molds (truncated, 12 × 12 × 20 mm) were purchased from Sigma-Aldrich, Saint Louis, MO, USA. Histology-grade solvents, Eosin G or Y (alcoholic solution 0.5%) and Masson’s Trichrome staining kit were purchased from DiaPath S.p.A., Martinengo, Italy, while QA-Agarose low melting point was obtained from MP Biomedical, Santa Ana, CA, USA. Harris hematoxylin, phosphate buffer saline (PBS) tablets, bovine serum albumin (BSA), SuperFrost Plus glass slides and cover slides were purchased from VWR International, LLC, Radnor, PA, USA. Trifluoroacetic acid (TFA), Antihuman CD68 antibody and AlexaFluor 568 goat anti mouse secondary antibody were purchased from Life Technologies-Invitrogen, Carlsbad, CA, USA. LC-MS grade water was purchased from Thermo Fisher Scientific, Rockford, IL, USA, while ITO coated glass slides for MALDI were obtained from Bruker Daltonics, Billerica, MA, USA. Paraformaldehyde 4% solution was purchased from Alfa Aesar, Haverhill, MA, USA.

### 3.2. Tissue Collection

Samples were collected from the Vascular Surgery Unit of the Pisa University Hospital. Clinical data were acquired from medical records, according to the Declaration of Helsinki. All subjects gave written informed consent to participate to the study. The ethical approval for this study was granted by the Ethics Commission of Tuscany Region (Protocol 37200, Comitato Etico Regionale per la Sperimentazione Clinica della Regione Toscana, Area Vasta Nord Ovest, approved on the 26 June 2019). Patients were classified as symptomatic if any neurological symptom linked to areas in the carotid-perfused territory occurred in a period of six months before surgery [13]. Patients received carotid endarterectomy if eligible according to the European Society of Vascular Surgery 2017 guidelines [13]. In particular, symptomatic patients with stenosis between 50% and 99% were eligible for surgery. Asymptomatic patients were considered eligible if the stenosis was between 60% and 99%, the patient’s life expectancy was higher than 5 years and one or more imaging characteristics associated with an increased risk of ipsilateral stroke were present [13]. The plaques were evaluated by a pathologist and histologically classified according to the American Heart Association (AHA, Dallas, TX, USA) scheme [53,54]. Plaque features of vulnerability were evaluated based on the thickness of the fibrous cap and on the plaque’s histological features [55]. Patient information is summarized in Table 3, and the histopathological evaluation of each plaque is provided in Supplementary Material 1 (including Figures S1–S6 and Table S1). Extended patient information is provided in Supplementary Material 4. Carotid plaques were collected after scheduled or emergency endarterectomy surgery, rinsed in physiological solution and immediately frozen on dry ice at −80 °C. Samples were stored at −80 °C until analysis.

### 3.3. Sample Preparation

Carotid plaques were transversally cut at −80 °C to expose the center of the plaque and embedded in a solution of 2% low melting point agarose. Embedding molds were kept in an ethanol–dry ice bath to limit tissue degradation and lipid diffusion during the embedding process. Tissue sections of 8 µm were cut using a Leica CM1950 cryostat (Leica, Wetzlar, Germany) and thaw mounted onto glass slides for histological and immunofluorescence assessment. Five glass slides carrying a duplicate of the tissue section were prepared for each plaque. Consecutive tissue sections of 12 µm thickness were cut and mounted onto poly-L-lysine coated ITO conductive slides for MALDI MSI. Two ITO slides were prepared for each sample, carrying two tissue sections each.

### 3.4. MALDI MSI Data Acquisition

ITO slides were thawed under vacuum for 15 min before matrix application. Consecutive sections were sprayed with norharmane (7 mg/mL, CHCl3:MeOH 70:30) and 2,5-dihydroxybenzoic acid (DHB, 30 mg/mL in MeOH:H2O 70:30, 0.2% TFA) using a SunCollect (SunChrom, Friedrichsdorf, Germany) spraying system (4 bar, 50 mm z-axis height). Both matrices were applied in 8 layers (2 layers at 5 µL/min followed by 6 layers at 10 µL/min). All tissue sections were dried under vacuum for 15 min prior to data acquisition. MALDI MSI was performed using an EP-MALDI source [56] (Spectroglyph, LLC., Kennewick, WA, USA) equipped with a 349 nm laser (Spectra-Physics, Santa Clara, CA, USA), coupled with an Orbitrap QExactive Plus (Thermo Scientific, Rockford, IL, USA). The laser was operated at 1.65 A and 500 Hz while source pressure was 7.2 Torr and pixel size was 30 × 30 µm. Spectra were acquired in positive-ion mode in the range 150–2000 *m/z* at 70,000 resolving power. The position file was aligned to the raw file using Image Insight (v. 0.1.0.11550, Spectroglyph, LLC).

### 3.5. Histological Staining

The tissue sections were stained with hematoxylin and eosin (H&E) after MSI data acquisition. Briefly, any residual MALDI matrix was removed using ethanol washes (2X 30 s each) followed by rehydration and H&E staining. Two consecutive tissue sections were stained with Masson’s Trichrome; the sections were fixed in Bouin’s solution for 1 h at 60 °C and then stained using Masson’s Trichrome kit (Diapath, Martinengo, Italy). High resolution optical images of the histologically stained tissues were recorded using an Aperio CS2 scanner at 40× magnification (Aperio Technologies Inc., Vista, CA, USA) and processed using Aperio ImageScope (v 12.2.2.5015, Aperio Technologies Inc.).

### 3.6. Immunofluorescence

Immunofluorescence was used to highlight VSMCs and macrophages using Monoclonal Anti-Actin α-SMA and antihuman CD68 antibodies, respectively. Tissue sections were fixed with 4% paraformaldehyde and membranes permeabilized with 0.2% Triton X-100 or 0.5% Tween 20 for α-SMA and CD68 immunofluorescence, respectively. Nonspecific staining was blocked with a 2% BSA solution. Tissues were incubated with the primary antibodies (1:800 dilution for α-SMA and 1:50 dilution for CD68) for 2 h at room temperature in the dark. AlexaFluor 568 goat antimouse secondary antibody was added (1:500 dilution) and incubated for 1 h at room temperature in the dark. Slides were then mounted using a DAPI-containing mounting medium and stored at 4 °C in the dark. Immunofluorescence images were acquired using an OLYMPUS Fluoview FV3000 system (Shinjuku, Tokyo, Japan). DAPI and secondary antibody channels were excited with a 405 nm and a 561 nm lasers, respectively. Brightfield images were acquired alongside the immunofluorescence images. Images were acquired at 4× magnification and selected fields were confirmed at 20× magnification. Images were exported from the Olympus FV315-SW software to ImageJ [57] for processing.

### 3.7. Histological Annotation

Histological and immunofluorescence images were used to annotate the tissue sections. Seven regions were annotated: collagen, hemorrhage, lipid-necrotic core, calcification, CD68 positive macrophages, α-SMA positive VSMCs of the media layer (outer VSMCs) and α-SMA positive VSMCs that migrated into the intima layer (inner VSMCs). The H&E image was cropped, resized and co-registered to the MALDI image using the MATLAB command *imregister*, affine mode. Co-registration parameters were tuned using the ImageRegistrationApp [58]. The borders of the annotated regions were imported into MATLAB and converted into masks. The same geometrical transformation applied to the H&E image was applied to each region mask to align it to the MALDI image.

### 3.8. MSI Data Preprocessing 

The raw spectra produced by the Orbitrap system during the MSI experiments were first converted into mzXML using RawConverter [59]. The MSI datacube was then extracted from the mzXML files and position files using a modified version of the script ORBIIMAGEmzXML2Tricks (v. 0.10, G. Eijkel). Datacubes were imported into MATLAB R2019b (MathWorks, Natick, MA, USA) for processing and analysis. In order to focus all subsequent data analysis steps on molecular signals that originated from the tissue, the spatial correlation between the tissue area and the distribution of each molecular ion was calculated. Only molecular ions with a correlation coefficient greater than 0.1 were retained. The selected molecular features were deisotoped using an in-house coded script and the intensity values of pixels outside the tissue area set to zero. MSI datasets from all tissue sections were combined into a merged datacube, keeping only those molecular ions common to all tissue samples. The merged datacube was TIC normalized and bright spots with an intensity greater than the 99.9th percentile were removed.

### 3.9. MSI Data Analysis

Merged MALDI images were weakly denoised using an edge-preserving [60] total variation minimizing Chambolle algorithm [61] (λ = 0.1, 1 iteration). Principal component analysis (PCA) and k-means clustering analysis were performed to evaluate the performance of each matrix in recapitulating the plaque’s histology. A Venn diagram was used to compare DHB and norharmane lipid profiles, using an *m/z* tolerance of 0.005 and was displayed using the web tool InteractiVenn [62]. Partial least squares (PLS) regression was performed to determine lipid signals characteristic of symptomatic plaques for each region. To avoid systematic bias arising from the different sizes of the regions in different patient samples PLS regression was performed using randomly selected pixels from each patient’s region-of-interest (ROI); the number of pixels used for the analysis corresponded to the greatest number that could be selected from all patient’s ROIs. PLS was performed in MATLAB using the *plsregress* function. The number of the model components for each region was chosen using a weight randomization test [63]. Characteristic features that discriminated between symptomatic and asymptomatic plaques were defined as the lipid ions with a variable importance in the projection (VIP) greater than one [64] with a loading on the first PLS axis exceeding the 90th percentile of the positive (increased in symptomatic) or negative (increased in asymptomatic) values. The first PLS axis was chosen since it is the axis that maximizes the covariance between the response and the predictors. 

The random selection of pixels used in the PLS analysis can introduce sampling bias due to the stochastic nature of random sampling. To guard against such bias, we repeated the random sampling and the PLS analysis three times, considering as dysregulated only those ions consistently identified by all the three replicates (Supplementary Material 5). Lipid ions were assigned on the basis of mass accuracy using METLIN [65] (Δppm ≤ 5 ppm) and are reported using the lipid species level notation [66]. An average spectrum for the lipid-necrotic core, macrophage-rich, and inner and outer VSMCs regions was produced by averaging the randomly selected spectra used for the PLS analysis. Hierarchical cluster analysis (Euclidean distance, average linkage) was then performed to compare how the lipid profiles of the different regions compared. Figures were finalized using Inkscape (v.1.0.1, Chino Hills, CA, USA).

## 4. Conclusions

Atherosclerotic plaques exhibit high heterogeneity. Spatially resolved techniques provide a better insight into the biology of atherosclerotic plaques by enabling the analysis to focus on specific actors within specific regions of the plaque. Here we set up a pipeline including sample preparation, MALDI MSI acquisition, histology and immunofluorescence for accurate region definition, annotation transfer to the MALDI MSI data, and regionwise multivariate data analysis. The approach allowed the regionwise comparison across multiple atherosclerosis patient samples. We applied the pipeline to a preliminary dataset to investigate the regiospecific differences in the lipid composition of symptomatic and asymptomatic plaques. It was found that the lipid species of macrophage-rich regions and inner VSMCs (close to the fibrotic cap) are associated with plaque outcome. In particular, macrophage-rich regions from symptomatic plaques showed an increase in sphingomyelins, a reduction of which has been linked to plaque stabilization in animal models [43]. Outer VSMCs in symptomatic patients showed an increase in cholesterol and cholesteryl esters, indicative of an increased lipid uptake. The proposed MALDI MSI pipeline is thus a promising tool to investigate ex vivo lipid markers of plaque vulnerability focusing on specific plaque regions.

## Figures and Tables

**Table 1:** Dysregulated lipids in the macrophage region of symptomatic plaques. Species with a VIP score greater than one and with a loading on the first PLS axis exceeding the 90th percentile of the positive or negative values are reported.

Matrix	Dysregulation in Symptomatic	*m/z*	Δppm	Adduct	Assignment
DHB	↑	369.35	5	[M+H-H2O]+	Cholesterol
DHB	↑	520.34	0	[M+H]+	LPC(18:2);
DHB	↑	542.32	3	[M+Na]+	LPC(18:2)
DHB	↑	544.34	0	[M+H]+	LPC(20:4)
DHB	↑	544.34	0	[M+H-H2O]+	PC(20:2)
DHB	↑	666.48			
DHB	↑	671.575	1	[M+H]+	CE(20:5)
DHB	↑	671.575	1	[M+Na]+	CE(18:2), zymosteryl oleate, 16:1 Stigmasteryl ester, 16:2 Sitosteryl ester
DHB	↑	711.54	1	[M+Na]+	Etn-1-P-Cer(22:1), SM(33:1), PE-Cer(36:1)
DHB	↑	723.54	1	[M+Na]+	SM(34:2), PE-Cer(37:2)
DHB	↑	725.555	2	[M+Na]+	SM(34:1), PE-Cer(37:1)
DHB	↑	741.53			
DHB	↑	807.635	0	[M+Na]+	SM(40:2)
DHB	↑	833.65	0	[M+Na]+	SM(42:3)
Nor.	↑	723.54	1	[M+Na]+	SM(34:2), PE-Cer(37:2)
Nor.	↑	835.67			
DHB	↓	697.475	4	[M+Na]+	PA(34:1)
DHB	↓	723.49	4	[M+Na]+	PA(36:2)
DHB	↓	756.55	1	[M+Na]+	PC(32:0), PE(35:0)
DHB	↓	780.55	1	[M+Na]+	PC(34:2), PE(37:2)
DHB	↓	782.565	2	[M+Na]+	PC(34:1), PE(37:1)
DHB	↓	804.55	1	[M+Na]+	PC(36:4), PE(39:4)
DHB	↓	806.565	2	[M+Na]+	PC(36:3), PE(39:3)
DHB	↓	808.58	3	[M+Na]+	PC(36:2), PE(39:2)
DHB	↓	832.58	3	[M+Na]+	PC(38:4), PE(41:4)
Nor.	↓	758.57	0	[M+H]+	PC(34:2), PE(37:2)
Nor.	↓	758.57	0	[M+H-H2O]+	PS(O-36:0)
Nor.	↓	782.57	0	[M+H]+	PC(36:4), PE(39:4)
Nor.	↓	782.57	0	[M+H-H2O]+	PS(O-38:3), PS(P-38:2)
Nor.	↓	808.585	0	[M+H]+	PC(38:5), PE(41:5)
Nor.	↓	808.585	0	[M+H-H2O]+	PS(O-40:4), PS(P-40:3)
Nor.	↓	808.585	2	[M+Na]+	PC(36:2), PE(39:2)

**Table 2:** Dysregulated lipids in the inner VSMCs region of symptomatic plaques. Species with a VIP score greater than one and with a loading on the first PLS axis exceeding the 90th percentile of the positive or negative values are reported.

Matrix	Dysregulation in Symptomatic	*m/z*	Δppm	Adduct	Assignment
DHB	↑	369.35	5	[M+H-H_2_O]^+^	Cholesterol
DHB	↑	671.575	1	[M+H]^+^	CE(20:5)
DHB	↑	671.575	1	[M+Na]^+^	CE(18:2), zymosteryl oleate, 16:1 Stigmasteryl ester, 16:2 Sitosteryl ester
DHB	↑	687.55	4	[M+Na]^+^	TG(38:1)
DHB	↑	725.555	2	[M+Na]^+^	SM(34:1), PE-Cer(37:1)
DHB	↑	741.53			
DHB	↑	758.57	0	[M+H]^+^	PC(34:2), PE(37:2)
DHB	↑	780.55	1	[M+Na]^+^	PC(34:2), PE(37:2)
DHB	↑	782.565	2	[M+Na]^+^	PC(34:1), PE(37:1)
DHB	↑	796.525	0	[M+Na]^+^	PE(P-40:7),1-(8-[3]-ladderane-octanoyl)-2-(8-[3]-ladderane-octanyl)-sn-glycerophosphoethanolamine
DHB	↑	798.54	1	[M+Na]^+^	PE(P-40:6)
DHB	↑	808.58	3	[M+Na]^+^	PC(36:2), PE(39:2)
Nor.	↑	780.555	1	[M+H]^+^	PC(36:5), PE(39:5)
Nor.	↑	780.555	4	[M+Na]^+^	PC(34:2), PE(37:2)
Nor.	↑	808.585	0	[M+H]^+^	PC(38:5), PE(41:5)
Nor.	↑	808.585	0	[M+H-H_2_O]^+^	PS(O-40:4), PS(P-40:3)
Nor.	↓	808.585	2	[M+Na]+	PC(36:2), PE(39:2)
DHB	↓	697.475	4	[M+Na]^+^	PA(34:1)
DHB	↓	773.505	1	[M+H-H_2_O]^+^	all-trans-nonaprenyl diphosphate
DHB	↓	778.605			
DHB	↓	832.58	3	[M+Na]^+^	PC(38:4), PE(41:4)
DHB	↓	837.68	2	[M+Na]^+^	SM(42:1)
DHB	↓	946.615			
Nor.	↓	723.54	1	[M+Na]^+^	SM(34:2), PE-Cer(37:2)
Nor.	↓	835.67	4	[M+Na]^+^	SM(42:2)

**Table 3:** Patients and plaques characteristics.

Patient	Age	Sex	Symptoms	Plaque AHA Classification
**1**	66	F	None	Type VI
**2**	84	F	None	Type Va
**3**	84	M	None	Type III
**4**	68	M	Transient ischemic attack	Type VI
**5**	75	M	Amaurosis	Type VI
**6**	73	F	Transient ischemic attack	Type VI

## Data Availability

The mass spectrometry proteomics data have been deposited to the ProteomeXchange Consortium via the PRIDE [67] partner repository with the dataset identifier PXD023663.

## Supplementary Materials

The following are available online at https://www.mdpi.com/article/10.3390/metabo11040250/s1, Supplementary Material 1. Histologic characterization of atherosclerosis plaques. It contains Figures S1–S6 and Table S1; Supplementary Material 2. Ions of the merged datacube used and ions in common between the DHB and the norharmane dataset; Supplementary Material 3. Dysregulated lipids in the macrophage, inner VSMCs, outer VSMCs, lipid-necrotic core and collagen regions of symptomatic plaques; Supplementary Material 4. Extended patient information; Supplementary Material 5. Replicates of random pixel sampling of PLS analysis. Supplementary Material 6. List of features detected in each plaque and features detected only in symptomatic and asymptomatic plaques; Figure S1. Annotated histological image of Patient 1; Figure S2. Annotated histological image of Patient 2; Figure S3. Annotated histological image of Patient 3; Figure S4. Annotated histological image of Patient 4; Figure S5. Annotated histological image of Patient 5; Figure S6. Annotated histological image of Patient 6; Figure S7. PCA analysis of the patient series. First eight PCs of the MALDI images acquired with DHB and norharmane. Regions are shown in the last panel (black: lipid-necrotic core, cyan: VSMCs outer, green: VSMCs inner, orange: macrophages, yellow: collagen, brown: hemorrhage, purple: calcification); Figure S8. Percentage of pixels classified in each cluster in the (a) DHB and (b) norharmane images. Data refer to Figure 4; Figure S9 Hierarchical cluster analysis of the lipid-necrotic core, outer VSMC, inner VSMC and macrophage regions. Average spectra of the regions were used to perform a hierarchical cluster analysis for the norharmane dataset; Table S1. Presence of the different regions in the plaques.
