# Supplementary material for: Mass Spectrometry Imaging as a Tool to Investigate Region Specific Lipid Alterations in Symptomatic Human Carotid Atherosclerotic Plaques
Source: Metabolites. 2021 Apr 18;11(4):250. doi: 10.3390/metabo11040250 (PMC8073208; doi:10.3390/metabo11040250)
Supplement: Supplementary file 1 [file metabolites-11-00250-s001.zip › Supplementary Material 1.pdf]

Supplementary Material 1

Histologic characterization of atherosclerosis plaques, based on hematoxylin and eosin staining and on Masson's Trichrome staining; annotated hematoxylin and eosin stained tissues (orange: Macrophages, cyan: Outer VSMCs; green: Inner VSMCs; yellow: Collagen; black: Lipid-necrotic core; brown: Hemorrhage; purple: Calcification).

Patient 1

Type VI plaque (AHA); it is a likely thick (> 1 µm, i.e., 322 µm) cap fibroatheroma, with intra-plaque hemorrhage and calcified foci. The cap is not clearly visible in an area close to a calcification.

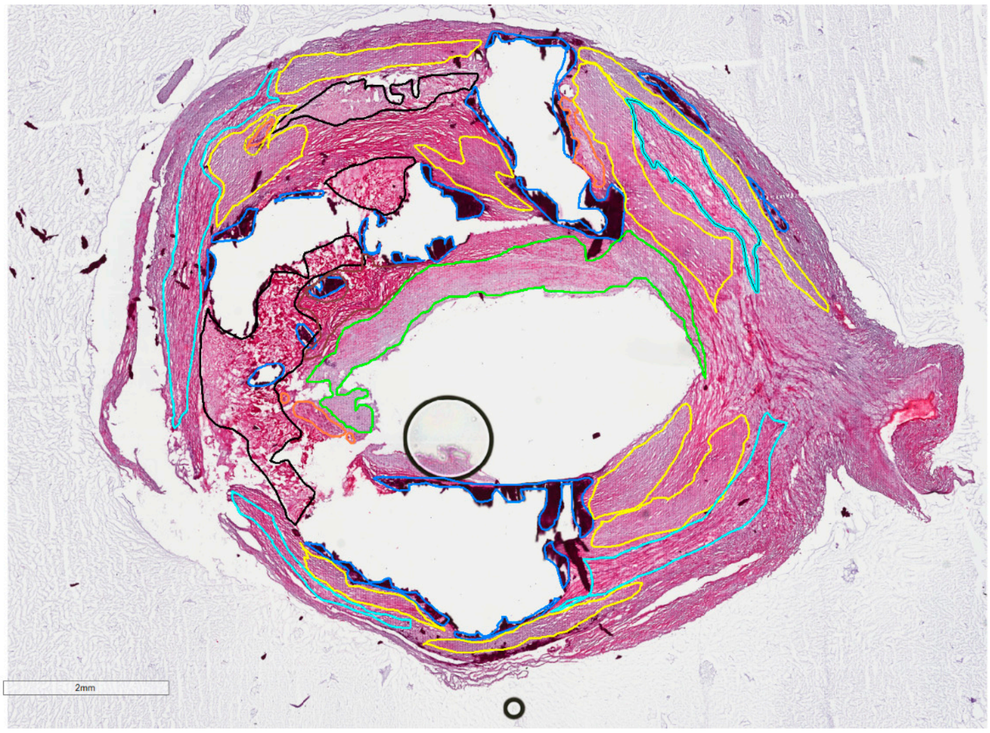

Figure S1. Annotated histological image of Patient 1.

Patient 2.

It is a Type Va plaque (AHA): fragments of an atherosclerotic plaque, recalling a fibroatheroma. Fibrous cap cannot be evaluated because of plaque fragmentation. It is characterized by a lipidic core and a fibrotic component; thrombus or intra-plaque hemorrhage are not evident (it could be a stable or non-vulnerable plaque based on the morphology, but such a definition is based on fibrous cap thickness > 165 µm).

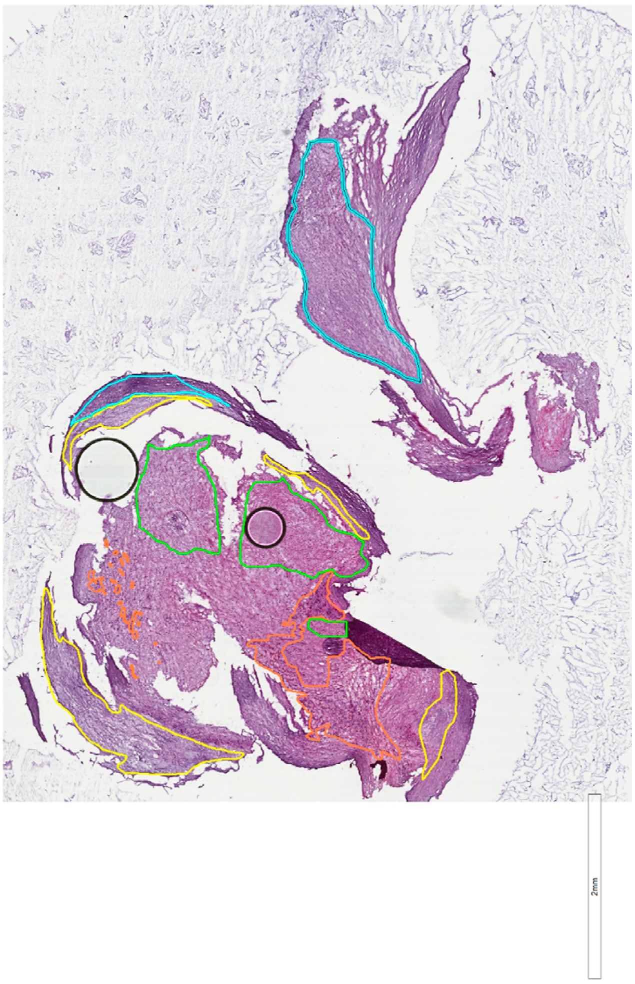

Figure S2. Annotated histological image of Patient 2.

Patient 3

Type III (intermediate) plaque (AHA): a plaque that is mainly constituted by fibrous (or fibro-muscular) tissue with a limited and predominantly intracellular lipidic component; little stenosis (non critical stenosis on this slide). The hemorrhagic and necrotic areas close to the lumen could represent fibrino-ematic stratifications. It looks like a stable (non-vulnerable) plaque.

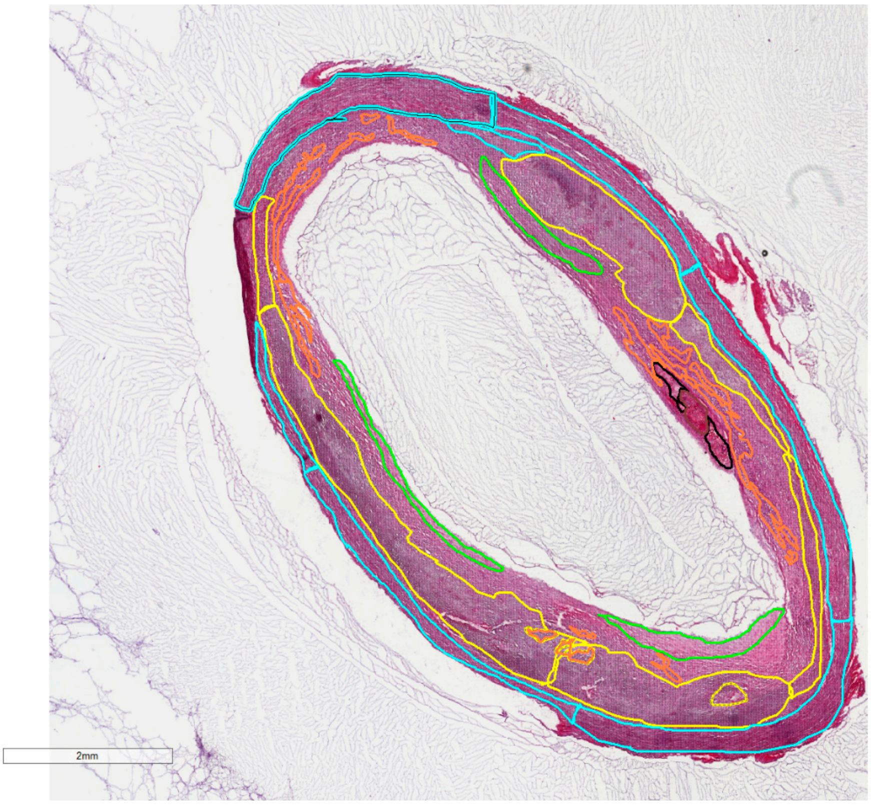

Figure S3. Annotated histological image of Patient 3.

Patient 4

Type VI plaque (AHA); it is a thick (> 165 μm, i.e., 175 μm) cap fibroatheroma, with intra-plaque hemorrhage.

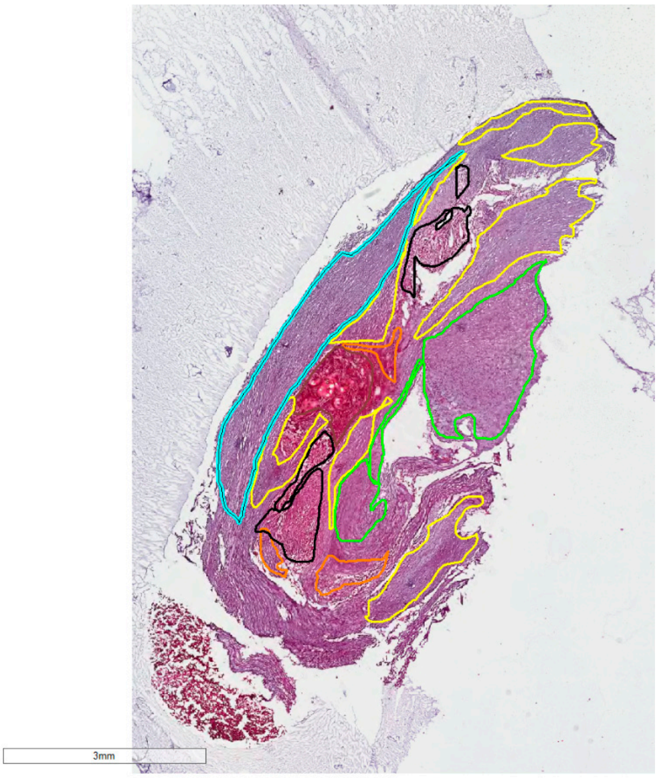

Figure S4. Annotated histological image of Patient 4.

Patient 5

Type VI plaque (AHA): a thin cap Fibroatheroma, apparently ulcerated with micro-thrombotic depositions and hemorrhage, focal calcification.

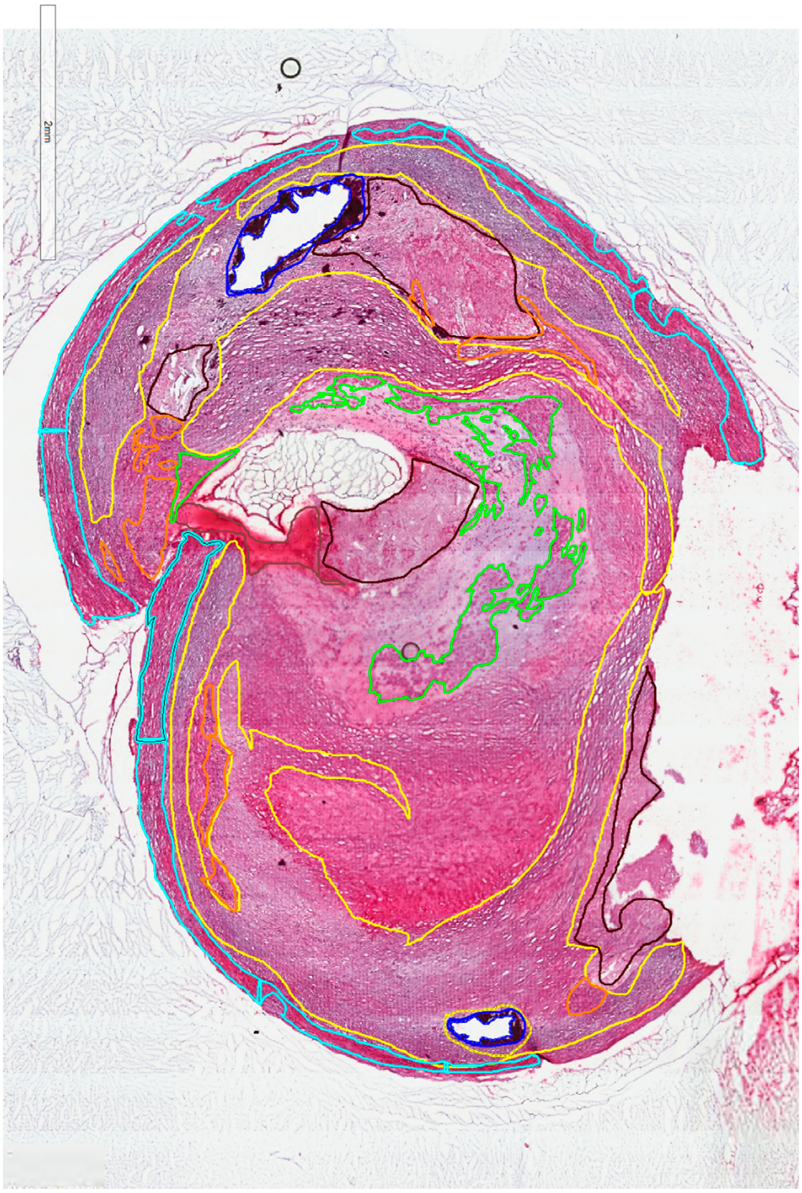

Figure S5. Annotated histological image of Patient 5.

Patient 6

It is a sub-occlusive Type VI plaque (AHA), i.e., a fibroatheroma with intra-plaque hemorrhage; it has a thick (> 165 μm, i.e., 340 μm) cap.

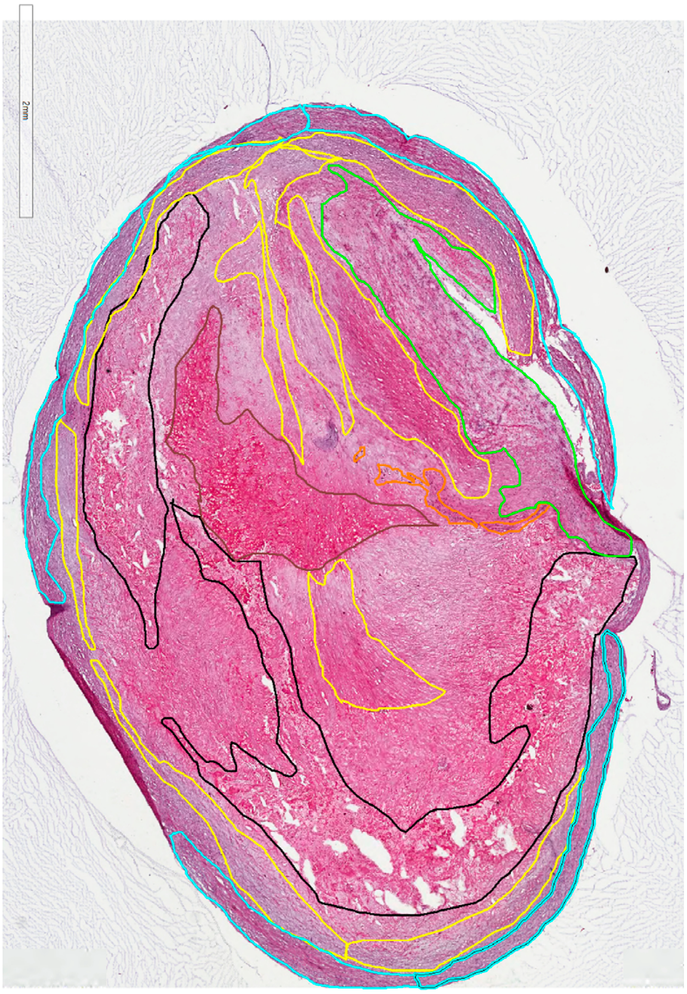

Figure S6. Annotated histological image of Patient 6.

Presence of the different regions in the plaques

Table S1. Presence of the different regions in the plaques.

Patient	1	2	3	4	5	6
Macrophages	X	X	X	X	X	X
VSMCs Inner	X	X	X	X	X	X
VSMCs Outer	X	X	X	X	X	X
Lipid-necrotic core	X		X	X	X	X
Collagen	X	X	X	X	X	X
Hemorrhage	X		X	X	X	X
Calcification	X				X	
